# Differential Functional Constraints Cause Strain-Level Endemism in *Polynucleobacter* Populations

**DOI:** 10.1128/mSystems.00003-16

**Published:** 2016-05-24

**Authors:** Naseer Sangwan, Iratxe Zarraonaindia, Jarrad T. Hampton-Marcell, Herbert Ssegane, Tifani W. Eshoo, Geeta Rijal, M. Cristina Negri, Jack A. Gilbert

**Affiliations:** a Biosciences Division, Argonne National Laboratory, Lemont, Illinois, USA; b Department of Surgery and Department of Ecology and Evolution, University of Chicago, Chicago, Illinois, USA; c Metropolitan Water Reclamation District of Greater Chicago, Cicero, Illinois, USA; d Energy Systems Division, Argonne National Laboratory, Lemont, Illinois, USA; Pacific Northwest National Laboratory

**Keywords:** metagenomics

## Abstract

The adaptation of bacterial lineages to local environmental conditions creates the potential for broader genotypic diversity within a species, which can enable a species to dominate across ecological gradients because of niche flexibility. The genus *Polynucleobacter* maintains both free-living and symbiotic ecotypes and maintains an apparently ubiquitous distribution in freshwater ecosystems. Subspecies-level resolution supplemented with metagenome-derived genotype analysis revealed that differential functional constraints, not geographic distance, produce and maintain strain-level genetic conservation in *Polynucleobacter* populations across three geographically proximal riverine environments. Genes associated with cofactor biosynthesis and one-carbon metabolism showed habitat specificity, and protein-coding genes of unknown function and membrane transport proteins were under positive selection across each habitat. Characterized by different median ratios of nonsynonymous to synonymous evolutionary changes (*dN*/*dS* ratios) and a limited but statistically significant negative correlation between the *dN*/*dS* ratio and codon usage bias between habitats, the free-living and core genotypes were observed to be evolving under strong purifying selection pressure. Highlighting the potential role of genetic adaptation to the local environment, the two-component system protein-coding genes were highly stable (*dN*/*dS* ratio, < 0.03). These results suggest that despite the impact of the habitat on genetic diversity, and hence niche partition, strong environmental selection pressure maintains a conserved core genome for *Polynucleobacter* populations.

**IMPORTANCE** Understanding the biological factors influencing habitat-wide genetic endemism is important for explaining observed biogeographic patterns. *Polynucleobacter* is a genus of bacteria that seems to have found a way to colonize myriad freshwater ecosystems and by doing so has become one of the most abundant bacteria in these environments. We sequenced metagenomes from locations across the Chicago River system and assembled *Polynucleobacter* genomes from different sites and compared how the nucleotide composition, gene codon usage, and the ratio of synonymous (codes for the same amino acid) to nonsynonymous (codes for a different amino acid) mutations varied across these population genomes at each site. The environmental pressures at each site drove purifying selection for functional traits that maintained a streamlined core genome across the Chicago River *Polynucleobacter* population while allowing for site-specific genomic adaptation. These adaptations enable *Polynucleobacter* to become dominant across different riverine environmental gradients.

## Abstract (precis)

Understanding the biological factors influencing habitat-wide genetic endemism is important for explaining observed biogeographic patterns. *Polynucleobacter* is a genus of bacteria that seems to have found a way to colonize myriad freshwater ecosystems and by doing so has become one of the most abundant bacteria in these environments. We sequenced metagenomes from locations across the Chicago River system and assembled *Polynucleobacter* genomes from different sites and compared how the nucleotide composition, gene codon usage, and the ratio of synonymous (codes for the same amino acid) to nonsynonymous (codes for a different amino acid) mutations varied across these population genomes at each site. The environmental pressures at each site drove purifying selection for functional traits that maintained a streamlined core genome across the Chicago River *Polynucleobacter* population while allowing for site-specific genomic adaptation. These adaptations enable *Polynucleobacter* to become dominant across different riverine environmental gradients.

## INTRODUCTION

The absence of geographic barriers, combined with oligotrophic conditions, can lead to inter- and intrapopulation-level competition within microbial communities (1). In addition, strain-level ecological adaptation, and hence radiation of the functional potential of a single species, enables some populations to dominate (arbitrarily defined here as >1 to 10% relative abundance) across ecosystems (2). The ubiquity of a single species (ecotype) could be explained by its genetic variance in fitness across a range of environments. Thus, ecological specialists (stenoecious) evolve in environments that are relatively homogeneous in space and time, whereas ecological generalists (euryoecious) evolve in environments with higher ecological diversification levels. Previous studies have used amplicon sequencing (e.g., 16S rRNA) to reveal this habitat-specific differentiation of strain-level genotypes (3). However, the interhabitat- and intrahabitat-wide impact of *in situ* functional constraints on the genetic evolution and ecology of dominant taxa can only be determined by population-level genomic analysis (4, 5).

*Polynucleobacter necessarius* is a planktonic freshwater bacterium in the family *Burkholderiaceae*. Taxa within this species have a small genome size and >99% identical 16S rRNA gene sequences (6). Currently, *P. necessarius* comprises two subspecies, (i) *P. necessarius* subsp. *asymbioticus*, and (ii) *P. necessarius* subsp. *necessarius. P. necessarius* subsp*. asymbioticus* comprises an ecologically cosmopolitan group of strains with a broad range of relative abundances extending from <1 to 70% (average, 20%) of the total bacterioplankton in freshwater aquifers (7). Strains associated with *P. necessarius* subsp. *necessarius* are obligate symbionts of ciliates (8). Despite detailed comparative genomic analysis of the cultured representatives (e.g., asymbiotic QLW-P1DMWA-1 and the endosymbiont STIR1 [9]), environmental *Polynucleobacter* populations remain uncharacterized.

On the basis of 16S rRNA gene amplicon analysis and deeply sequenced (>10 Gb) metagenomic data assembled across seven sites in three different regions of the Chicago Area Waterway System (CAWS), we provide evidence, using *Polynucleobacter*, for the “ubiquity-by-diversification” theory, which states that intrataxon lineage-specific adaptations to local environmental pressures (metabolic and physical) lead to a ubiquitous distribution and high relative abundance of the adapted taxon (2). These sites were chosen to represent regional variability in a land use-type context, physicochemical parameters, and proximity to wastewater treatment plants. For clarity, we use the term genotype throughout this report for *de novo*-assembled populations (10, 11).

## RESULTS

### Amplicon sequencing reveals significant correlations between microbial community structure and both geography and physicochemical parameters.

**FIG 1:**
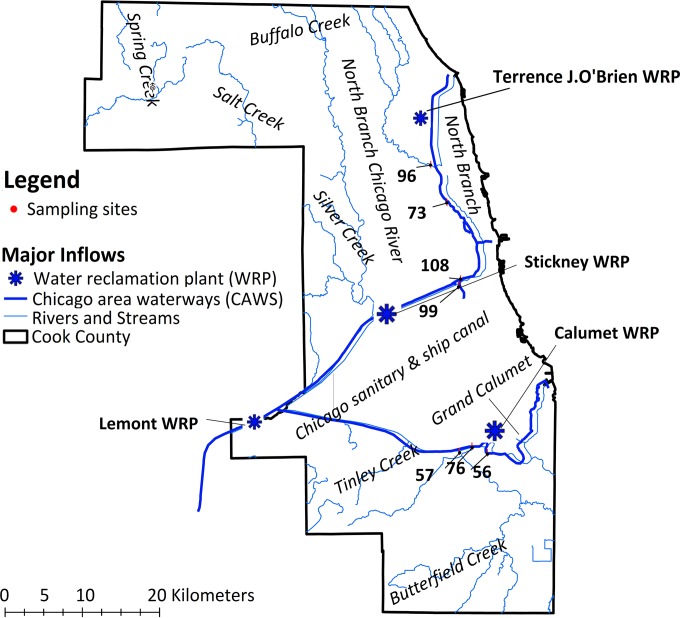
Geographic locations along the CAWS of the sampling sites used in this study.

**FIG 2:**
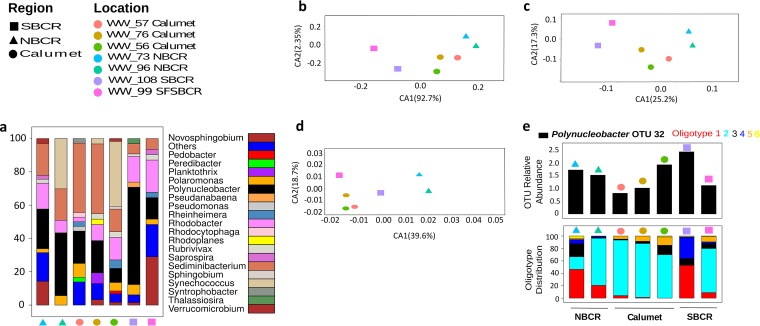
Taxonomic characterization and ordination at the OTU and sub-OTU levels between groups. (a) Genus-level differential abundance analysis (ANOVA, *P* < 0.001) between groups. (b) Unweighted UniFrac distance beta diversity analysis of 16S rRNA OTUs (97%). (c) Unweighted UniFrac distances for just the *Polynucleobacter* taxa (*n* = 70; 16S rRNA V4; 97% OTUs). (d) Phylogeny independent ordination of Bray-Curtis dissimilarity for just *Polynucleobacter* oligotypes (*n* = 6). (e) Relative abundance of *Polynucleobacter* OTU32 (top), which was significantly different between regions, and oligotype distribution of *Polynucleobacter* OTU32 (bottom) across the three regions. Different symbols and colors represent WRP and sampling locations, respectively.

Microbial communities were analyzed by 16S rRNA V4 amplicon sequencing, generating 12,871 to 208,721 reads per sample (Table 1) that, after quality control and rarefaction, clustered into 16,511 operational taxonomic units (OTUs; 97% nucleotide similarity). Using an arbitrary cutoff of three reads per OTU, only 461 OTUs were selected for all of the subsequent downstream analysis. Microbial community composition was significantly different between geographic locations (Fig. 1) with significantly differentiated microbial community structures (ADONIS partial *R*^2^ = 0.43, *P* = 0.037, unweighted UniFrac). Microbial diversity (the number of OTUs) was significantly positively correlated with total dissolved solids (*R*^2^ = 0.507, *P* < 0.01), total Kjeldahl nitrogen (*R*^2^ = 0.27, *P* < 0.01), and ammonia (*R*^2^ = 0.37, *P* < 0.01). *Proteobacteria*, *Cyanobacteria*, *Actinobacteria*, and *Bacteroidetes* dominated across all of the samples (see Fig. S1A in the supplemental material). The genera *Rhodobacter*, *Novosphingobium*, *Synechococcus*, *Sediminibacterium*, and *Polynucleobacter* were differentially abundant across all three regions (analysis of variance [ANOVA], Tukey-Kramer *post hoc* test, and Bonferroni correction, *P* < 0.05; Fig. 2a). Microbial community beta diversity was less variable within regions than between regions (Fig. 2b and c). Geographic distance (kilometers) had a weak but significant positive correlation with the distribution of total bacterial diversity (*R*^2^ = 0.3, *P* = 0.013; see Fig. S2A in the supplemental material).

10.1128/mSystems.00003-16.1 Figure S1  Method-independent validation of *Polynucleobacter* genetic predominance. (A) 16S rRNA gene-based (OTUs, 97% identity) taxonomic analysis of total microbial diversity (phylum). Samples are grouped and colored according to the WRP names. (B) Nonmetric multidimensional scaling plots generated from Bray-Curtis distances based on metagenome contigs (NBC Classifier) (i) and MetaPhlAn taxonomy analysis (ii). Rarified abundance of *Polynucleobacter* is shown for the same ordination with greater bubble size and the contour lines indicating abundance. Download Figure S1, TIF file, 2.2 MB. Copyright © 2016 Sangwan et al. 2016 Sangwan et al. This content is distributed under the terms of the Creative Commons Attribution 4.0 International license.

10.1128/mSystems.00003-16.2 Figure S2  Phylogenetic and genetic differentiation across geographic distance. For each sample pair, geographic distance (kilometers) was plotted against the phylogenetic (A to C) and genotypic (core genome) (D) distances. Bray-Curtis distances (based on 16S rRNA OTUs, 97% identity) were computed with the vegan package. Pairwise Fst values were computed across the core genomes by using single nucleotide polymorphism profile comparisons implemented in the Poopulation2 software. The Mantel test was performed to identity the correlation between two matrices. Geographic distance had a very small but significant impact on the total diversity. However, there was no significant impact of distance on the OTU-, sub-OTU-, and core genome-based genetic differentiation of *Polynucleobacter* populations. Download Figure S2, TIF file, 1.8 MB. Copyright © 2016 Sangwan et al. 2016 Sangwan et al. This content is distributed under the terms of the Creative Commons Attribution 4.0 International license.

**TABLE 1:** Geographic locations, summary characteristics, and metadata of CAWS metagenomes

Feature	WW76	WW56	WW57	WW96	WW73	WW99	WW108
Maximum contig size (bp)	37,724	42,876	38,779	15,324	26,559	92,129	36,280
No. of 16S rRNA reads	27,668	12,871	23,198	208,721	14,683	21,772	23,885
Region	Calumet	Calumet	Calumet	NBCR	NBCR	SBCR	SBCR
Temp (ºC)	24.3	24.1	23.5	13.4	17.1	21.1	21.6
Shannon diversity index	5.50782	5.11079	5.23254	5.15931	5.23976	4.74986	5.5954
Pielou’s evenness	0.74616	0.7205	0.70941	0.71528	0.71988	0.67439	0.7607
Latitude	41.6575	41.6503	41.6517	41.9743	41.9324	41.8386	41.8459
Longitude	−87.6411	−87.6171	−87.6606	−87.7061	−87.6829	−87.664	−87.6606
Dissolved oxygen concn (mg/liter)	6.7	6.5	6.9	8.1	7.5	7.8	5.6
pH	7.34	8.01	6.64	7.95	7.42	7.06	6.5
Ammoniacal nitrogen concn (mg/liter)	0.11	0.12	0.19	0.11	0.1	0.7	0.49
Total Kjeldahl nitrogen concn (μg/ml)	0.6	0.7	0.7	0.5	0.8	2.03	0.6
SO_4_ concn (mg/liter)	53.11	29.39	23.73	60.37	50.41	52.38	32.91
Alkalinity	137	259	108	165	159	164	116
Chloride concn (mg/liter)	62	70	63	235	63	171	62
Fluoride concn (mg/liter)	0.38	0.15	0.14	0.36	0.47	0.59	0.43
Total organic C concn (mg/liter)	3.5	2.1	1.9	4.6	5.1	5.9	3.2
Phenol concn (mg/liter)	4.1	3.1	3.9	4.1	4	2.8	3.2

*Polynucleobacter* 16S rRNA sequences were resolved to the strain level by oligotyping (12), and the oligotypes had a pattern of reduced beta diversity within sites in the same region similar to that found between regions (Fig. 2d and e). Oligotyping was performed on the dominant *Polynucleobacter* OTU (OTU32), resulting in six oligotypes across all seven sites. Oligotype 2 was extremely dominant and was found at all of the sites except WW99 (South Branch Chicago River [SBCR]). (For clarity, we use the term SBCR for the WW99 and WW108 sites throughout this paper.) The beta diversity pattern of these *Polynucleobacter* oligotypes showed a significant positive correlation with the concentration of ammonia (Biology Environ, UniFrac *R*^2^ = 0.7, *P* < 0.01), as did the abundance of oligotype 2 (*R*^2^ = 0.56, *P* < 0.05). Geographic localization (kilometers) had no significant correlation with either OTU or oligotype distribution (see Fig. S2B and C in the supplemental material). However, the beta diversity pattern of physicochemical measurements (see Fig. S3 in the supplemental material) was similar to the taxonomy analysis (Fig. 1b), suggesting that physicochemical factors, and hence local adaptation, shape *Polynucleobacter* diversity.

10.1128/mSystems.00003-16.3 Figure S3  Physicochemical-measurement-based principal-component analysis of microbial communities assembled across all three WRPs. Download Figure S3, TIF file, 1.1 MB. Copyright © 2016 Sangwan et al. 2016 Sangwan et al. This content is distributed under the terms of the Creative Commons Attribution 4.0 International license.

### Metagenomic annotation and method-independent validation of *Polynucleobacter* abundance.

Fourteen metagenomes (two technical replicates per sample site) were sequenced, producing numbers of quality-trimmed reads ranging from 25 to 100 million (Table 1). 16S rRNA gene rarefaction curves (see Fig. S4A in the supplemental material) and abundance-based weighted coverage (13) and assembly analysis {e.g., N50 values [North Branch Chicago River (NBCR), 412.50 ± 52.50; SBCR, 437 ± 7; Calumet, 494.33 ± 45.86]; Table 1} gave similar sequence complexity trends across the three regions. NBCR samples had the lowest functional gene diversity and richness (H = 4 ± 0.2; J = 0.73 ± 0.13), in contrast to Calumet site samples (H = 6.03 ± 0.003; J = 0.91 ± 0.32) and SBCR (H = 5.2 ± 0.42; J = 0.86 ± 0.001). NBCR also had the smallest average genome size (AGS) (see Fig. S4B in the supplemental material), which could indicate oligotrophy through ecological adaptation (14). MetaPhAln analysis and metagenome assembly (contigs assigned to taxa) were used to validate the 16S rRNA amplicon analysis. *Polynucleobacter* bacteria were abundant at all of the sites (see Fig. S1B in the supplemental material). The beta-diversity trend of unassembled metagenomic data, annotated to known functional genes (see Fig. S4C in the supplemental material), was similar to the taxonomic analysis (Fig. 2b), suggesting that functional adaptations underlie taxon-specific differentiation across regions (15).

10.1128/mSystems.00003-16.4 Figure S4  Downstream analysis reveals similar sequence diversities but different AGS patterns across WRPs. (A) 16S rRNA gene-based rarefaction plots were generated with the vegan package. (B) AGS calculations were performed with the micobeCensus program (27). *Polynucleobacter* taxa had a smaller AGS than the total microbial community. (C) Functional-information-based principal-component analysis of microbial communities assembled across all three WRPs. (D) Functional-information (enzyme)-based principal-component analysis of *Polynucleobacter* bins assembled across all three WRPs. Download Figure S4, TIF file, 2.4 MB. Copyright © 2016 Sangwan et al. 2016 Sangwan et al. This content is distributed under the terms of the Creative Commons Attribution 4.0 International license.

Discounting core metabolic functions (e.g., energy metabolism, transcription, and translation), the relative abundance of aminobenzoate degradation, amino acid biosynthesis (valine, leucine, and isoleucine), mismatch repair, phosphotransferase system, and polycyclic aromatic hydrocarbon degradation were significantly different among the regions (ANOVA, Tukey-Kramer *post hoc* test, and Bonferroni correction, *P* < 0.05; see Table S1 in the supplemental material). Enzyme-level functional analysis (BLASTX-based KEGG mapping) highlighted the TonB-linked outer membrane protein (SusC/RagA family), leucine/isoleucine/valine transporter permease, and amine transporter categories as differentially abundant across all three regions. The SBCR and NBCR regions had the most divergent functional profiles (*R*^2^ = 0.76; two-sided Welch *t* test, *P* < 0.05), while Calumet (*n* = 3) and SBCR (*n* = 2) had significantly similar functional profiles (*R*^2^ = 0.96, *P* < 0.005).

10.1128/mSystems.00003-16.5 Table S1  Multigroup (ANOVA, Bonferroni correction) analysis of total community metabolism. Categories were selected according to a cutoff of *P* < 0.05. Download Table S1, XLS file, 0.04 MB. Copyright © 2016 Sangwan et al. 2016 Sangwan et al. This content is distributed under the terms of the Creative Commons Attribution 4.0 International license.

### Population-level genetic differentiation across *in situ Polynucleobacter* cohorts.

Corresponding to previously published culture-dependent and -independent studies (4), our 16S rRNA gene amplicon- and shotgun metagenomics-based analysis highlighted that *in situ Polynucleobacter* populations follow the ubiquity-by-diversification theory, whereby lineage-specific ecological preferences result in niche separation (Fig. 2c to e) (2). Intrataxon-specific AGS variations (see Fig. S4B in the supplemental material) and distance decay analysis (see Fig. S2A in the supplemental material) suggest that local functional constraints (physical environment) are fundamental for lineage-specific genetic adaptation. To test this hypothesis at the level of the population genome, we employed nucleotide composition-based (tetranucleotide frequency usage and G+C content [percent]) genotype reconstruction methods to bin *Polynucleobacter* contigs (representing *in situ* strains) across each sample (see Table S2 in the supplemental material). Marker gene copy number variation (CNV) analysis revealed that sites WW73 (NBCR) and WW76 (Calumet) had the maximum (*n* = 12) and minimum (*n* = 3) numbers of *Polynucleobacter* species, respectively. Individual-read-based AGS variation analysis (see Fig. S4B in the supplemental material) revealed that *Polynucleobacter* cohorts had a smaller AGS (NBCR, 1.9 ± 0.19 Mb; Calumet, 2.7 ± 0.23 Mb; SBCR, 2.8 ± 0.16 Mb) than the total community (i.e., NBCR, 2.6 ± 0.11 Mb; Calumet, 3.4 ± 0.26 Mb; SBCR, 4.0 ± 0.19 Mb). *Polynucleobacter* population bin size (total) varied across metagenome samples, which positively correlated with sequencing depth (Table 1). WW76 and WW57 (Calumet) had the greatest and least sequencing depths (100 million and 25 million reads, respectively) and *Polynucleobacter* population bin sizes (7.1 and 1.9 Mb, respectively).

10.1128/mSystems.00003-16.6 Table S2  Genetic features of *Polynucleobacter* populations across CAWS sites. Download Table S2, XLS file, 0.02 MB. Copyright © 2016 Sangwan et al. 2016 Sangwan et al. This content is distributed under the terms of the Creative Commons Attribution 4.0 International license.

**FIG 3:**
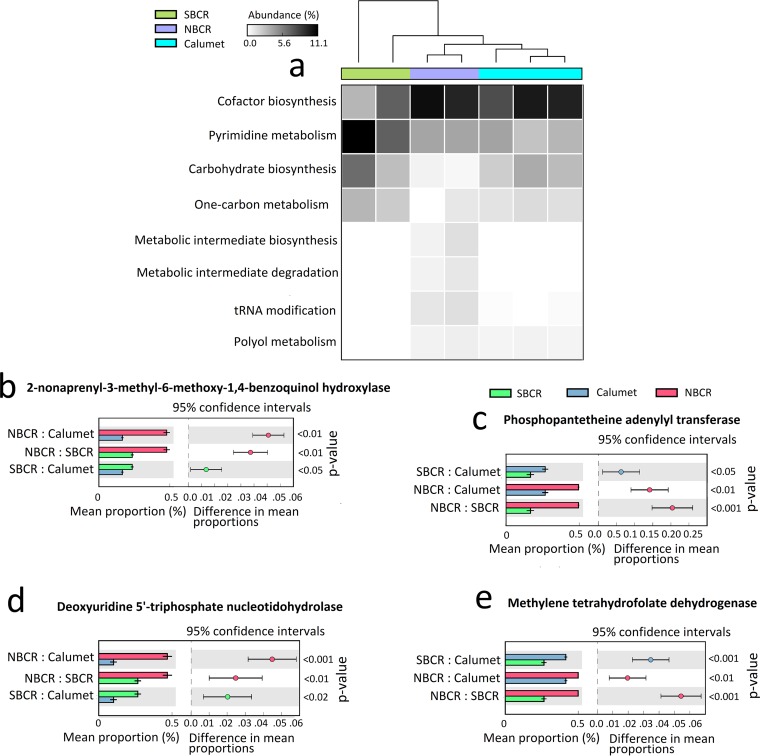
Habitat (WRP)-wide differences in the relative abundance of metabolic pathways and enzymes assembled across *Polynucleobacter* population bins. (a) ANOVA-based comparative analysis of metabolic pathways enriched across *Polynucleobacter* population bins. Welch *t*-test-based comparative analysis of key enzymes involved in cofactor biosynthesis (b and c), pyrimidine metabolism (d), and one-carbon metabolism (e). The FDR was corrected at *P* < 0.05 (Bonferroni correction).

*P. necessarius* subspecies can maintain free-living and endosymbiont modes of life (8, 9). In order to study the *in situ* population-level evolutionary dynamics of these two modes of life (endosymbiotic and free living), we compared the whole-genome sequences of the available symbiotic (*P. necessarius* subsp. *necessarius* STIR1) (16) and free-living taxa (*P. necessarius* subsp. *asymbioticus* QLW-P1DMWA-1 and betaproteobacterium CB [17]). The *Polynucleobacter* population bins (protein-coding genes) from the present study were thus demarcated into endosymbiont (*n* = 205)-, free-living organism (*n* = 336)-, and core (*n* = 1,579)-specific genetic repertoires (see Tables S3 and S4 in the supplemental material). Pairwise orthologous gene identification categorized 205 protein-coding genes as endosymbiont specific, which contrasts with the 105 genes identified previously (9). The majority of the samples showed a greater abundance of free-living organism (48 to 71%)- and core genome (49 to 71%)-specific genotypes than endosymbiont-specific gene content (10 to 24%) (see Table S3 in the supplemental material). Consistent with previous reports (4), the *Polynucleobacter* population core genome has the potential for urea (Pnuc_1190 to Pnuc_1202), inorganic sulfur (Pnuc_1476 to Pnuc_1494), and nitrogen (Pnuc_0987 to Pnuc_1003) metabolism. However, pathway- and enzyme-level comparisons (intergroup) of the core genome revealed significant differences in energy metabolism across *Polynucleobacter* ecotypes (Fig. 3a). Pantothenate (vitamin B_5_, Pnec_0171)- and ubiquinone-based cofactor biosynthesis (Pnec_0171) was highly abundant (ANOVA, *P* < 0.05) across NBCR and Calumet ecotypes (Fig. 3b and c), in contrast to SBCR samples. However, SBCR ecotypes had a greater abundance of genes involved in pyrimidine metabolism (Fig. 3a and d, dUMP biosynthesis from dCTP, Pnec_0171). Similarly, one-carbon metabolism (tetrahydrofolate interconversion, methylenetetrahydrofolate dehydrogenase, Pnec_0171) showed significant variations across all three regions (Fig. 3a and e). These habitatwise differential patterns of substrate utilization and of energy metabolism demonstrate mechanisms of local environmental adaptation, as hypothesized for cultured *Polynucleobacter* strains (4).

10.1128/mSystems.00003-16.7 Table S3  Protein-coding genes identified in the endosymbiont, free-living, and core genotypes of *Polynucleobacter* populations assembled across CAWS locations. The total number of genes in each category is in parentheses. Download Table S3, XLS file, 0.02 MB. Copyright © 2016 Sangwan et al. 2016 Sangwan et al. This content is distributed under the terms of the Creative Commons Attribution 4.0 International license.

10.1128/mSystems.00003-16.8 Table S4  Multigroup analysis of the functional repertoire of core, free-living, and endosymbiont genotypes of *Polynucleobacter* populations. Download Table S4, XLS file, 0.04 MB. Copyright © 2016 Sangwan et al. 2016 Sangwan et al. This content is distributed under the terms of the Creative Commons Attribution 4.0 International license.

The trends in beta diversity between samples for the functional annotations of the *Polynucleobacter* bins (protein-coding genes annotated to metabolic enzymes) were similar to the trends observed in the taxonomic analysis (see Fig. S4D in the supplemental material). *Polynucleobacter* bins had pseudogene profiles more closely related to those of free-living subspecies (*P. necessarius* subsp. *asymbioticus* QLW-P1DMWA-1) than to those of endosymbionts (*P. necessarius* subsp. *necessarius* STIR1). Also, distance decay analyses of the core genome-based allele frequency (pairwise Fst index) suggested that geographic distance had no significant impact on the intrapopulation-level genetic differentiation of *Polynucleobacter*, unlike the genus-level 16S rRNA amplicon analysis, which suggests that genotypic studies provide higher-resolution investigations (see Fig. S2D in the supplemental material). This observation further highlights that, despite a potentially high dispersal rate, environmental selection has an important role in shaping the *in situ Polynucleobacter* community gene content.

### Effect of environmental selection on *Polynucleobacter* evolution.

**FIG 4:**
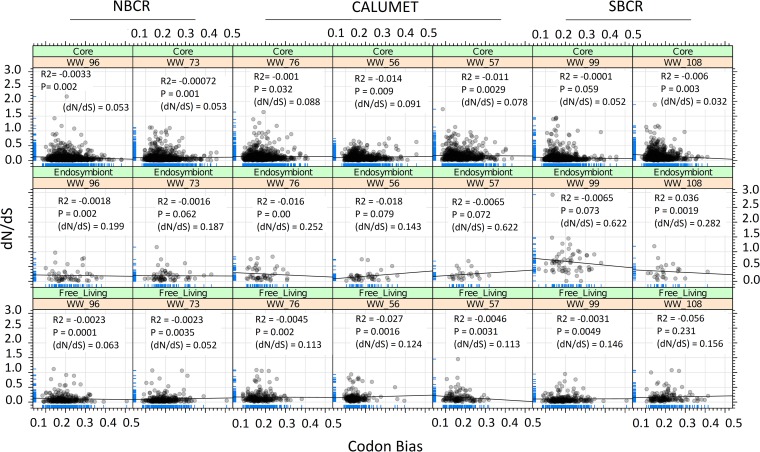
Coupling between natural selection (*y* axis) and CUB (*x* axis) validates the habitat-wide impact of *in situ* functional constraints across free-living-organism-, core-, and endosymbiont-specific gene contents of *Polynucleobacter* taxa. Reverse BLAST hit-based orthologous genes were used to perform pairwise *dN*/*dS* ratio and CUB analyses. The codon deviation coefficient was computed by methods explained in reference 41.

Population-level orthologous gene pairs (>300 bp) for endosymbiont (*n* = 205)-, free-living organism (*n* = 336)-, and core (*n* = 1,579)-specific gene contents were analyzed for natural selection patterns (ratio of nonsynonymous to synonymous evolutionary changes [*dN*/*dS* ratio]) across all seven sites. Highlighting the strength of purifying natural selection (*dN*/*dS* ratio, <1), the majority of genes had median *dN*/*dS* ratios ranging in 0.01 to 0.09, whereas fast-evolving (*dN*/*dS* ratio, >1) protein-coding genes were less prevalent (*n* = 1 to 8/site) across all of the groups (Fig. 4). Using BLASTX annotation results against the NCBI nr data set, the majority (>80%) of the positively selected genes (annotated; *dN*/*dS* ratio, >1) were assigned to the transferase and membrane transport enzyme-based categories. Interestingly, two putative horizontal gene transfer events (specifically, shikimate dehydrogenase from bacterium UASB14 and heme ABC transporter permease from *Pusillimonas*) were also observed to have higher *dN*/*dS* ratios (>1) in the core genotypes of WW76 (Calumet) and WW96 (NBCR) samples, respectively.

Recently, Ran et al. (18) revealed that patterns of correlation between the CUB (codon usage bias) and *dN*/*dS* ratio of orthologous genes from closely related genomes could be used as a metric to analyze lineage-specific environmental adaptation. In other words, the relationship between two aspects of selection (CUB and *dN*/*dS* ratio) can be used as a metric to highlight the importance of translation fine-tuning genetic adaptation to environment changes. In order to validate our hypothesis that strong *in situ* functional constraints are shaping the natural selection trends across *in situ Polynucleobacter* populations, orthologous protein pairs were analyzed to study the coupling between the *dN*/*dS* ratio and CUB. Interestingly, a limited but statistically significant (negative) correlation pattern was observed for the free-living and core genotypes (Fig. 4).

## DISCUSSION

Freshwater ecosystems have greater microbial AGSs than marine ecosystems (14, 19). These AGS differences indicate the selection pressure of local metabolic (functional) constraints on genomic adaptation. At the population level, the AGS variation may represent the *in situ* metabolic potential of a taxon. Specifically, an ecogenetically adapted generalist copiotroph should have a larger genome size than a specialized yet abundant oligotroph, but variation within a single-species population also suggests that AGS is significantly influenced by adaptation to local environmental conditions.

Using 16S rRNA amplicon and shotgun metagenomic sequencing, we have investigated the population ecology of the dominant freshwater bacterium *Polynucleobacter* across all three main regions of the CAWS, i.e., NBCR, SBCR, and Little Calumet River (LCR) Discounting methodological bias (e.g., amplification, sequencing quality, and coverage), this study suggests that *Polynucleobacter* bacteria are abundant across all three regions of the CAWS, suggesting that interhabitat strain-specific genetic adaptation may enable *Polynucleobacter* taxa to become ubiquitous in freshwater ecosystems (7).

The AGS of the *Polynucleobacter* assembled bins was smaller than that of the total community (see Fig. S4B in the supplemental material), suggesting that the genus *Polynucleobacter* is a specialized taxon. Geographic distance had no major role in shaping the interhabitat-level population dynamics of *Polynucleobacter* bacteria, as highlighted by the distance decay analysis of oligotyping-based beta diversity patterns and the genetic differentiation of the assembled core genome (Fst; see Fig. S2C and D in the supplemental material). Metabolic differences in energy metabolism, substrate utilization (Fig. 3), and the concentration of available nutrients (e.g., ammoniacal nitrogen) seem to have a stronger influence on the *Polynucleobacter* population structure. This variance in genetic fitness suggests that the ubiquity of *Polynucleobacter* strains results from stenoecious (specialist) genetic adaptations and not from euryoecious (generalist) adaptations.

Therefore, we hypothesize that *in situ* metabolic constraints were the primary reason for the genotypic diversity seen. The genome-wide influence of environmental selection (functional constraints) on the CAWS *Polynucleobacter* population was determined by using pairwise *dN*/*dS* ratios for orthologous genes. Habitatwise selection analysis of the core-, free-living-organism-, and endosymbiont-specific genotypes revealed that while the majority of genes were under purifying natural selection (median *dN*/*dS* ratios, 0.01 to 0.09), which is already known for most bacterial genomes (20), there was differential selection pressure for each genotype (free-living, core, and endosymbiont) for specific genes across each habitat (Fig. 4). Specifically, the core genes had the lowest median *dN*/*dS* ratio (Fig. 4) across each habitat. However, this observation is expected because a core gene codes for important metabolic enzymes and has a direct metabolic interaction with the environment (18; see also Table S4 in the supplemental material).

To further confirm that the different selection patterns were due to strong and diverse environmental selection (*in situ* functional constraints), we further analyzed the patterns of correlation between CUB and the *dN*/*dS* ratio across orthologous protein-coding genes. Interestingly, a weak but significant negative correlation was observed between the *dN*/*dS* ratio and CUB across core and free-living genotypes. Since we have used evolutionarily conserved (orthologous genes) and population-level genetic information for natural selection and CUB analysis, we assume that pairwise *dN*/*dS* ratio and CUB variations of these genes directly represent the strength of *in situ* functional constraints. The observation of habitat-wide differential correlation patterns between the *dN*/*dS* ratio and CUB supports our hypothesis that functional constraints cause and maintain the strain-level genetic endemism, and hence the genotypic diversity, of *Polynucleobacter* populations, and thus, loss of function would be deleterious to the organism.

## MATERIALS AND METHODS

### Site selection, sampling, and physicochemical analysis.

Figure 1 shows the seven sites selected for this study. The sites represent highly altered channelized streams of the CAWS, including the NBCR (sites WW73 and WW96), SBCR (sites WW108 and WW99), South Fork SBCR (known as Bubbly Creek), and the LCR (sites WW56, WW57, and WW76), which is not directly connected to the NBCR. Sites WW56 and WW76 are upstream and downstream of a major inflow into the CAWS from the Calumet Water Reclamation Plant (WRP), and site 73 is downstream of the Terrence J. O’Brien WRP, another major inflow. Sites WW96 and WW57 include tributaries that contribute flow to the CAWS. These sites constitute part of the current ambient water quality monitoring sampling stations of the Metropolitan Water Reclamation District (MWRD) of Greater Chicago. All locations were sampled monthly by surface grab sampling and analyzed for physicochemical parameters including pH, water temperature, alkalinity, total suspended solids, ammonia, nitrate, phosphorus, total metals, dissolved metals, cyanide, phenol, and fecal coliform bacteria, while organic priority pollutants and nonylphenols were sampled semiannually and quarterly, respectively (Table 1 shows sample parameters). Both pH and water temperature were measured at each site.

### 16S rRNA gene amplicon data analysis.

Paired-end reads were quality trimmed and processed for OTU clustering with the UPARSE pipeline (21), set at a 97% identity cutoff. High-quality (<1% incorrect bases) OTUs were assigned to various taxonomic levels by using the parallel_assign_taxonomy_rdp.py script from QIIME software (22). Multiple-sequence alignment and phylogenetic reconstruction were performed with PyNast and FastTree (22). The Phyloseq package (23) was used for detailed downstream analysis, e.g., alpha and beta diversity-based ordination on a rarefied abundance matrix. The OTU matrix was processed to remove OTUs containing fewer than three reads and rarified to the minimum numbers of reads present in the smallest library (11,083 reads). We used the oligotyping pipeline (12) to identify the sub-OTU-level differences across the *Polynucleobacter* taxon, i.e., one of the five most differentially abundant genera, i.e., *Rhodobacter*, *Synechococcus*, *Sediminibacterium*, *Polynucleobacter*, and *Novosphingobium*, as predicted by MetagenomeSeq (24).

### Quality filtering, coverage estimation, metagenome assembly, and annotation.

Paired-end metagenome reads were quality trimmed with nesoni (GitHub Victorian Bioinformatics Consortium) by using the following parameters: a minimum length of 75, a quality cutoff of 30, adapter trimming, and 0 ambiguous bases. Taxonomic and functional information was assigned to the individual metagenome reads with MetaPhlAn (25) and MGRAST (26), respectively. Individual-read-based functional annotations were used for functional diversity and richness estimation. Quality-trimmed metagenome reads were assembled into contigs with IDBA_UD (27) by using k-mer lengths ranging from 31 to 41. Metagenome contigs with lengths of <300 bp were excluded from further analysis. Metagenome contigs were assigned to various taxonomic levels by NBC Classifier (28). Average metagenome coverage and sequence diversity were computed for each sample with Nonpareil (13) set at default parameters. AGS was computed for each metagenome sample and *Polynucleobacter* bin with MicrobeCensus (29). FragGeneScan (30) was also used to predict the protein-coding genes across metagenome contigs. Functional annotation of individual metagenome reads and contigs (ORFs) was performed with paladin (GitHub) and prokka (31), respectively.

### Genotype binning and population-level comparative genomics.

In order to understand the population-level dynamics (taxonomic, functional, and evolutionary) of *Polynucleobacter* across these sites, we focused our further assembly efforts to bin population genomes (genotypes, not individual genomes) for this taxon. Tetranucleotide frequency usage and G+C content values (percent) were computed for each metagenome contig with 2TBinning (32). Contigs were clustered into bins with hierarchical agglomerative clustering performed with an interprofile correlation cutoff (*R*^2^) of 0.9. Chimeric contigs, i.e., those that differed from the mean G+C content (percent) by more than 1 standard deviation, were removed from the individual population bin. *Polynucleobacter* bins were further screened (Nmer = 12) for the contaminants (assigned to different taxons) with NBC Classifier (28). Single-copy marker gene-based CNV analysis (33) was used to estimate the number of species across each bin. To predict the number of species across each site, single-copy genes were clustered at 97% identity. By using reference genomes pairwise, the orthologous gene prediction method (34) was used to demarcate *in situ Polynucleobacter* population gene contents into core-, free-living-organism-, and endosymbiont-specific genes. Pseudogenes were predicted across population genomes with GenePRIMP (35). Reconstructed population genomes were uploaded to the RAST server (36) for automated genome annotation. Fst calculations were performed for the core gene contents of *Polynucleobacter* populations across all seven metagenome samples with the PoPoolation2 software (37).

### Evaluation and validation of the influence of *in situ* functional constraints.

Pairwise selected orthologous protein-coding genes were aligned with Clustal W (38). Multiple-codon alignments were constructed from the corresponding aligned protein sequences with pal2nal script (39). Final alignments (stop codons removed) were processed for *dN*/*dS* ratio analysis with PAML (40). To further validate the influence of *in situ* functional constraints on the observed natural selection patterns, we processed the orthologous gene pairs by using codon bias variation. By methods explained in reference 41, the codon deviation coefficient was used as the measure of codon bias across orthologous gene pairs predicted across free-living, endosymbiont, and core genotypes. The mean value of two orthologous genes was used for the correlation analysis against *dN*/*dS* ratios.

### Statistical analysis.

All of the statistical analyses done in this study were performed in the R environment (R Development Core Team, 2012). Multigroup and two-group comparisons were performed by ANOVA (Tukey-Kramer *post hoc* test, effect size = Eta-squared and multiple-test correction by Storey’s false-discovery rate [FDR]) and Welch *t* test (two sided and multiple-test correction by Storey’s FDR), respectively. Beta diversity was analyzed by calculating distance decay with the vegdist package implemented in R (R Development Core Team, 2012). Briefly, Bray-Curtis similarity matrices were created from the OTU and oligotype data and Euclidean distance matrices were created from the distances between individual samples. The MetagenomeSeq package (24) was used to identify the differentially abundant taxons. The number of reads per kilobase per genome equivalent was also used to normalize and quantify the pathway and/or subsystem abundance from shotgun metagenomes as predicted by MGRAST (26).

### Conclusions.

Different *in situ* functional constraints cause and maintain a conserved core genome in *Polynucleobacter* populations. Observed patterns of coupling between the *dN*/*dS* ratio and CUB highlight that translational fine-tuning likely helps *Polynucleobacter* bacteria to adapt to subtle metabolic changes in the local environment. However, dominant taxa are known to have complex interspecies metabolic tradeoffs (5) that can influence their genetic evolution, and therefore, understanding the biological factors that influence this habitat-wide genetic conservation remains a challenge for future studies.

### Nucleotide sequence accession numbers.

The metagenome data obtained in this study have been uploaded to MG-RAST under project no. 7450 and accession numbers 4549281.3, 4549282.3, 4549324.3, 4549324.4, 4549328.3, 4549328.4, 4549326.3, 4549327.3, 4549334.3, 4549335.3, 4549338.3, 4549339.3, 4549392.3, and 4549393.3.
