# Supplementary figures and images for: Differential Functional Constraints Cause Strain-Level Endemism in Polynucleobacter Populations
Source: mSystems. 2016 May 24;1(3):e00003-16. doi: 10.1128/mSystems.00003-16 (PMC5069759; doi:10.1128/mSystems.00003-16)

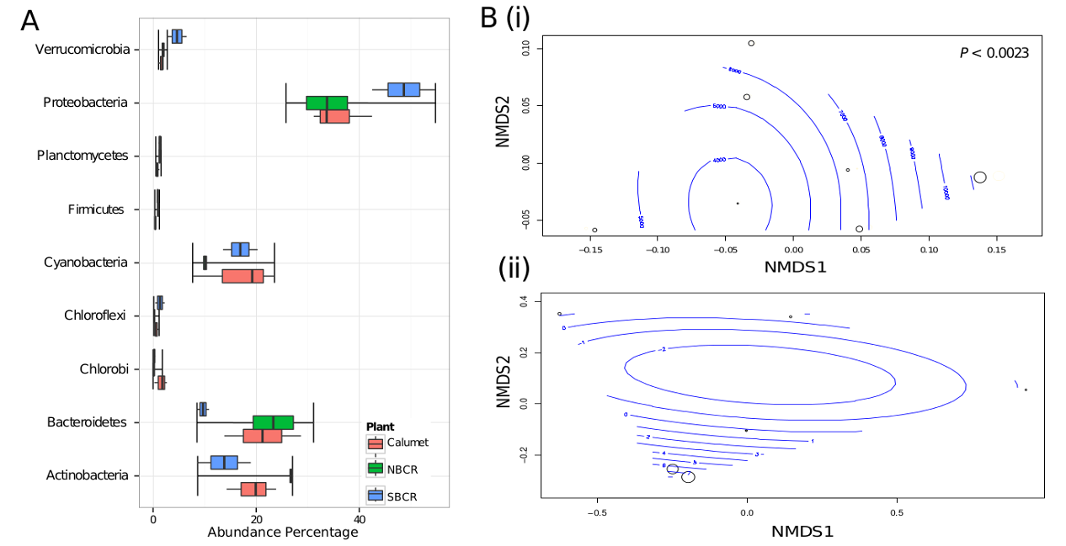

Supplement: Figure S1 [file sys003162027sf1.tif]

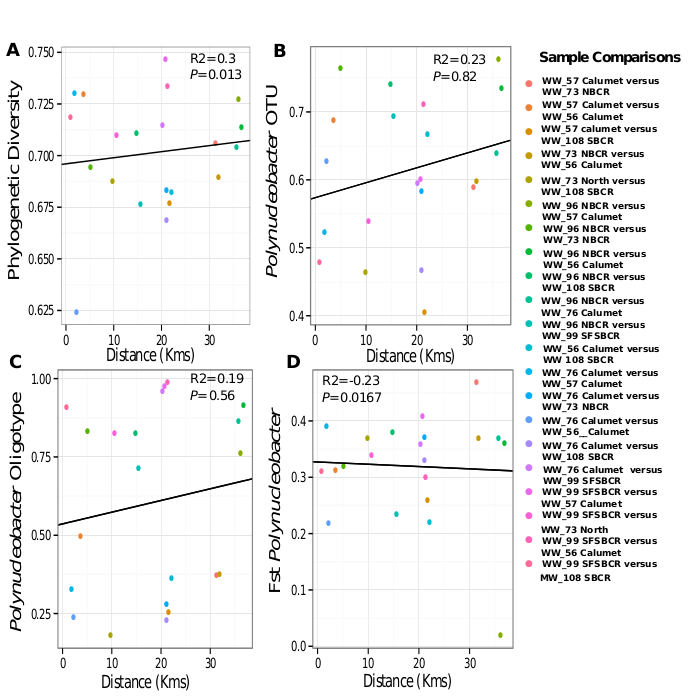

Supplement: Figure S2 [file sys003162027sf2.tif]

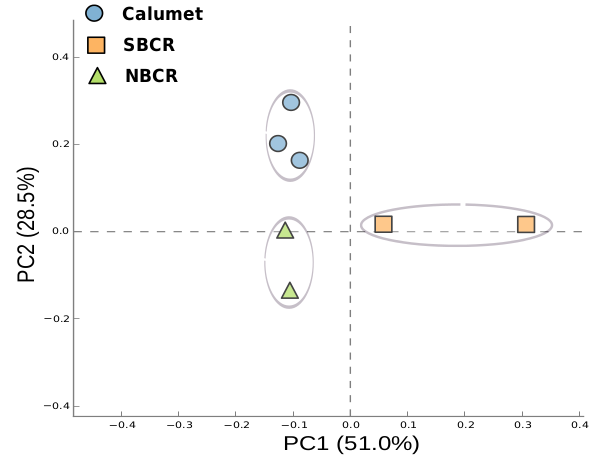

Supplement: Figure S3 [file sys003162027sf3.tif]

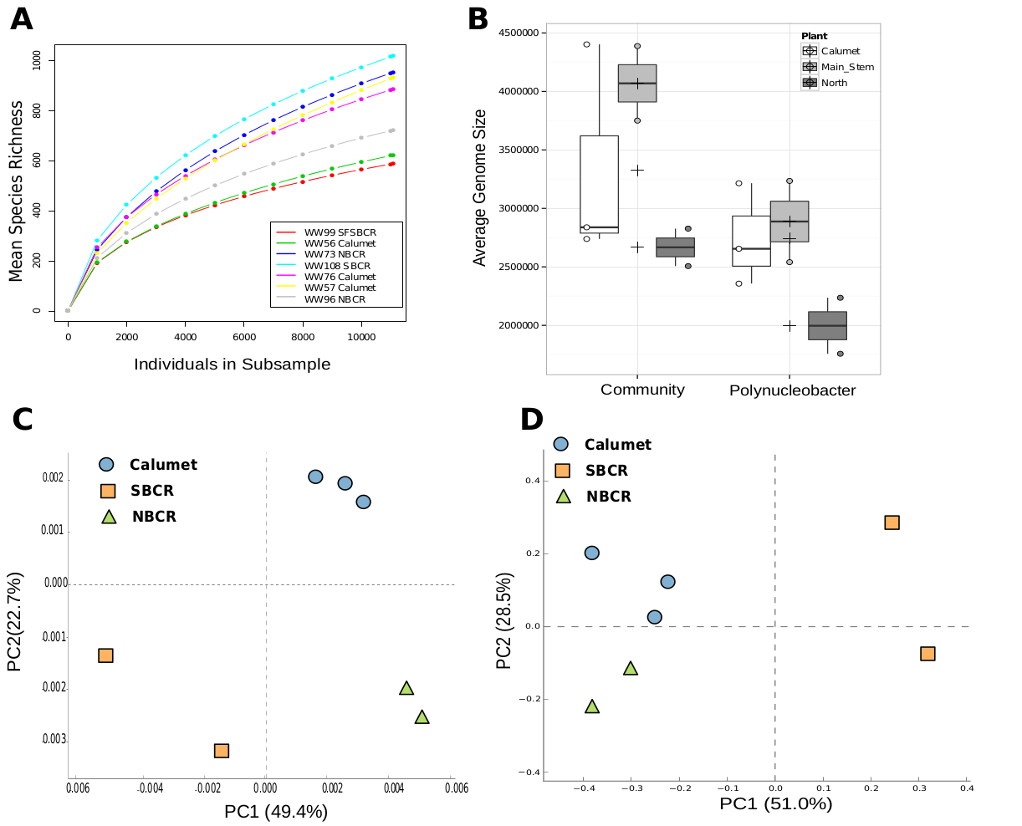

Supplement: Figure S4 [file sys003162027sf4.tif]
